# Supplementary material for: Deciphering the variation in cuticular hydrocarbon profiles of six European honey bee subspecies
Source: BMC Ecol Evol. 2024 Oct 28;24:131. doi: 10.1186/s12862-024-02325-z (PMC11520070; doi:10.1186/s12862-024-02325-z)
Supplement: Supplementary file 1 — Supplementary Material 1 [file 12862_2024_2325_MOESM1_ESM.docx]

# Supplementary material

Table S1: CHC profile of forager bees from different *A. mellifera* subspecies showing mean relative abundance (%) and standard deviation (sd) of all compounds. Mean and sd values are in bold for the cases a compound is present in only one subspecies.

| Compound | RI | *A. m. carnica* | | *A. m. iberiensis* | | *A. m. ligustica* | | *A. m. macedonica* | | *A. m. mellifera* | | *A. m. ruttneri* | |
| --- | --- | --- | --- | --- | --- | --- | --- | --- | --- | --- | --- | --- | --- |
|  |  | mean | sd | mean | sd | mean | sd | mean | sd | mean | sd | mean | sd |
| C17:2 | 1,659 | 0.000 | 0.000 | **0.037** | **0.040** | 0.000 | 0.000 | 0.000 | 0.000 | 0.000 | 0.000 | 0.000 | 0.000 |
| 8-C17:1 | 1,666 | 0.000 | 0.000 | 0.117 | 0.109 | 0.000 | 0.000 | 0.040 | 0.051 | 0.000 | 0.000 | 0.000 | 0.000 |
| C17 | 1,700 | 0.000 | 0.000 | 0.148 | 0.090 | 0.000 | 0.000 | 0.000 | 0.000 | 0.000 | 0.000 | 0.351 | 0.689 |
| C19:2 | 1,863 | 0.000 | 0.000 | **0.021** | **0.018** | 0.000 | 0.000 | 0.000 | 0.000 | 0.000 | 0.000 | 0.000 | 0.000 |
| 9-C19:1 | 1,875 | 0.164 | 0.222 | 0.365 | 0.355 | 0.000 | 0.000 | 0.212 | 0.135 | 0.163 | 0.179 | 0.000 | 0.000 |
| C19 | 1,900 | 0.000 | 0.000 | 0.382 | 0.115 | 0.126 | 0.193 | 0.035 | 0.051 | 0.922 | 1.344 | 0.999 | 1.149 |
| C20 | 2,000 | 0.000 | 0.000 | 0.038 | 0.031 | 0.000 | 0.000 | 0.000 | 0.000 | 0.140 | 0.054 | 0.131 | 0.061 |
| C21:2 | 2,065 | 0.000 | 0.000 | **0.020** | **0.019** | 0.000 | 0.000 | 0.000 | 0.000 | 0.000 | 0.000 | 0.000 | 0.000 |
| 9-C21:1 | 2,073 | 0.000 | 0.000 | 0.199 | 0.096 | 0.053 | 0.032 | 0.117 | 0.066 | 0.091 | 0.030 | 0.042 | 0.031 |
| C21 | 2,100 | 0.462 | 0.307 | 2.169 | 2.670 | 0.947 | 0.460 | 1.232 | 1.808 | 7.911 | 4.145 | 7.666 | 3.052 |
| C22:1 | 2,173 | 0.000 | 0.000 | 0.027 | 0.016 | 0.000 | 0.000 | 0.000 | 0.000 | 0.035 | 0.028 | 0.000 | 0.000 |
| C22 | 2,200 | 0.231 | 0.100 | 0.232 | 0.078 | 0.291 | 0.088 | 0.235 | 0.099 | 0.390 | 0.138 | 0.305 | 0.115 |
| C23:2 | 2,264 | 0.000 | 0.000 | 3.258 | 6.887 | 0.000 | 0.000 | 0.000 | 0.000 | 1.551 | 4.905 | 0.000 | 0.000 |
| 9-; 7-C23:1 | 2,271 | 2.330 | 0.904 | 2.557 | 0.804 | 3.128 | 0.870 | 2.112 | 1.311 | 3.124 | 0.548 | 6.194 | 7.084 |
| C23 | 2,300 | 22.271 | 6.827 | 8.712 | 3.011 | 19.051 | 5.173 | 15.350 | 7.051 | 14.868 | 2.453 | 12.367 | 5.246 |
| 11-MeC23 | 2,334 | 0.000 | 0.000 | 0.000 | 0.000 | 0.000 | 0.000 | 0.000 | 0.000 | **0.044** | **0.026** | 0.000 | 0.000 |
| 9-C24:1 | 2,371 | 0.103 | 0.057 | 0.108 | 0.064 | 0.753 | 1.879 | 0.161 | 0.110 | 0.000 | 0.000 | 0.248 | 0.207 |
| C24 | 2,400 | 0.651 | 0.195 | 0.542 | 0.341 | 0.770 | 0.200 | 0.660 | 0.238 | 0.485 | 0.074 | 0.430 | 0.113 |
| C25:2_1 | 2,455 | 0.000 | 0.000 | 0.000 | 0.000 | 0.000 | 0.000 | 0.000 | 0.000 | 0.000 | 0.000 | **0.248** | **0.191** |
| 4-MeC24 | 2,460 | 0.000 | 0.000 | **0.044** | **0.071** | 0.000 | 0.000 | 0.000 | 0.000 | 0.000 | 0.000 | 0.000 | 0.000 |
| C25:2_2 | 2,465 | 0.280 | 0.120 | 0.239 | 0.116 | 0.779 | 0.885 | 0.000 | 0.000 | 0.145 | 0.154 | 0.000 | 0.000 |
| 9-; 7-C25:1 | 2,471 | 3.680 | 1.291 | 2.343 | 0.504 | 4.975 | 1.352 | 4.008 | 2.133 | 3.944 | 1.512 | 3.202 | 2.028 |
| C25 | 2,500 | 23.436 | 9.057 | 14.301 | 3.752 | 19.671 | 3.011 | 19.900 | 3.858 | 13.483 | 2.381 | 15.080 | 2.412 |
| 13-; 11-MeC25 | 2,533 | 0.000 | 0.000 | 0.066 | 0.038 | 0.000 | 0.000 | 0.046 | 0.041 | 0.192 | 0.094 | 0.000 | 0.000 |
| 4-MeC25 | 2,561 | 0.000 | 0.000 | **0.017** | **0.015** | 0.000 | 0.000 | 0.000 | 0.000 | 0.000 | 0.000 | 0.000 | 0.000 |
| 9-C26:1 | 2,574 | 0.000 | 0.000 | 0.405 | 1.015 | 0.000 | 0.000 | 0.114 | 0.081 | 0.000 | 0.000 | 0.000 | 0.000 |
| C26 | 2,600 | 0.465 | 0.074 | 1.260 | 1.321 | 0.596 | 0.104 | 0.607 | 0.200 | 0.679 | 0.157 | 0.531 | 0.107 |
| C27:2_1 | 2,643 | 0.000 | 0.000 | **0.040** | **0.026** | 0.000 | 0.000 | 0.000 | 0.000 | 0.000 | 0.000 | 0.000 | 0.000 |
| 4-MeC26 | 2,660 | 0.000 | 0.000 | **0.041** | **0.034** | 0.000 | 0.000 | 0.000 | 0.000 | 0.000 | 0.000 | 0.000 | 0.000 |
| 9-C27:1 | 2,671 | 0.695 | 0.280 | 1.243 | 0.536 | 1.276 | 0.282 | 1.561 | 0.591 | 2.476 | 0.381 | 0.900 | 0.682 |
| 7-C27:1 | 2,678 | 0.205 | 0.082 | 0.830 | 0.412 | 0.402 | 0.140 | 0.410 | 0.184 | 1.585 | 0.449 | 0.910 | 1.218 |
| C27 | 2,700 | 13.644 | 4.010 | 18.993 | 3.968 | 12.207 | 2.933 | 11.978 | 2.224 | 14.714 | 3.667 | 11.788 | 3.734 |
| 13-; 11-MeC27 | 2,727 | 0.233 | 0.071 | 0.305 | 0.123 | 0.383 | 0.139 | 0.314 | 0.103 | 0.355 | 0.094 | 0.307 | 0.092 |
| 3-MeC27 | 2,772 | 0.000 | 0.000 | 0.000 | 0.000 | 0.000 | 0.000 | **0.324** | **0.446** | 0.000 | 0.000 | 0.000 | 0.000 |
| C28 | 2,800 | 0.348 | 0.176 | 0.908 | 0.218 | 0.565 | 0.193 | 0.358 | 0.172 | 0.667 | 0.554 | 0.000 | 0.000 |
| C29:2_1 | 2,847 | 0.000 | 0.000 | 0.000 | 0.000 | 0.000 | 0.000 | **0.084** | **0.050** | 0.000 | 0.000 | 0.000 | 0.000 |
| 4-MeC28 | 2,860 | 0.000 | 0.000 | 0.629 | 1.093 | 0.000 | 0.000 | 0.000 | 0.000 | 0.000 | 0.000 | 0.152 | 0.107 |
| 10-; 9-; 8-; 7-C29:1 | 2,875 | 0.777 | 0.517 | 3.357 | 1.366 | 0.833 | 0.758 | 2.968 | 3.747 | 3.096 | 0.457 | 0.958 | 0.448 |
| C29 | 2,900 | 9.402 | 4.936 | 14.411 | 3.342 | 8.897 | 1.982 | 7.669 | 2.702 | 7.637 | 1.703 | 4.374 | 2.234 |
| 15- ; 13-MeC29 | 2,928 | 0.204 | 0.074 | 0.299 | 0.150 | 0.252 | 0.146 | 0.223 | 0.115 | 0.239 | 0.101 | 0.164 | 0.075 |
| 11, 17-diMeC29 | 2,958 | 0.000 | 0.000 | 0.000 | 0.000 | 0.000 | 0.000 | **0.069** | **0.098** | 0.000 | 0.000 | 0.000 | 0.000 |
| 4-MeC29 | 2,959 | 0.000 | 0.000 | **0.085** | **0.132** | 0.000 | 0.000 | 0.000 | 0.000 | 0.000 | 0.000 | 0.000 | 0.000 |
| 3-MeC29 | 2,967 | 0.000 | 0.000 | **0.119** | **0.162** | 0.000 | 0.000 | 0.000 | 0.000 | 0.000 | 0.000 | 0.000 | 0.000 |
| 10-; 9-; 8-; 7-C30:1 | 2,976 | 0.000 | 0.000 | 0.499 | 0.542 | 0.000 | 0.000 | 0.126 | 0.110 | 0.000 | 0.000 | 0.000 | 0.000 |
| C30:1 | 2,983 | 0.000 | 0.000 | **0.015** | **0.025** | 0.000 | 0.000 | 0.000 | 0.000 | 0.000 | 0.000 | 0.000 | 0.000 |
| C30 | 3,000 | 0.130 | 0.095 | 0.452 | 0.119 | 0.519 | 0.248 | 0.293 | 0.120 | 0.290 | 0.080 | 0.791 | 1.479 |
| C31:2_1 | 3,051 | 0.000 | 0.000 | 0.173 | 0.126 | 0.000 | 0.000 | 0.058 | 0.044 | 0.147 | 0.057 | 0.000 | 0.000 |
| C31:2_2 | 3,057 | 0.111 | 0.103 | 0.763 | 0.433 | 0.000 | 0.000 | 0.167 | 0.133 | 0.202 | 0.179 | 0.000 | 0.000 |
| 4-MeC30 | 3,064 | 0.000 | 0.000 | 1.973 | 2.238 | 0.000 | 0.000 | 0.298 | 0.675 | 0.000 | 0.000 | 0.000 | 0.000 |
| 10-; 9-; 8-C31:1 | 3,075 | 5.316 | 2.532 | 6.246 | 2.320 | 4.336 | 0.966 | 5.918 | 2.588 | 4.582 | 1.799 | 8.951 | 4.672 |
| C31:1 | 3,087 | 0.000 | 0.000 | 0.000 | 0.000 | 0.000 | 0.000 | 0.000 | 0.000 | 0.000 | 0.000 | **0.693** | **0.531** |
| C31 | 3,100 | 5.403 | 3.771 | 4.147 | 2.741 | 5.685 | 1.676 | 6.559 | 2.288 | 3.865 | 1.657 | 3.859 | 1.550 |
| 15-; 13-MeC31 | 3,125 | 0.000 | 0.000 | **0.222** | **0.238** | 0.000 | 0.000 | 0.000 | 0.000 | 0.000 | 0.000 | 0.000 | 0.000 |
| 10-; 9-C32:1 | 3,173 | 0.183 | 0.092 | 0.278 | 0.148 | 0.000 | 0.000 | 0.968 | 0.966 | 0.380 | 0.430 | 1.452 | 1.408 |
| C32 | 3,200 | 0.000 | 0.000 | 0.179 | 0.206 | 0.000 | 0.000 | 0.286 | 0.150 | 0.000 | 0.000 | 0.000 | 0.000 |
| C33:2_1 | 3,249 | 0.000 | 0.000 | 0.000 | 0.000 | 0.000 | 0.000 | 1.248 | 0.761 | 1.110 | 2.307 | 0.905 | 0.668 |
| C33:2_2 | 3,257 | 0.000 | 0.000 | 1.009 | 0.374 | 1.246 | 0.444 | 0.000 | 0.000 | 0.000 | 0.000 | 0.000 | 0.000 |
| 10-C33:1 | 3,278 | 8.235 | 3.361 | 4.149 | 2.318 | 10.174 | 5.150 | 8.159 | 4.110 | 9.939 | 6.127 | 10.887 | 3.840 |
| C33 | 3,300 | 0.800 | 0.709 | 1.026 | 0.561 | 2.083 | 1.023 | 2.547 | 2.074 | 0.000 | 0.000 | 3.042 | 4.541 |
| C35:2_1 | 3,449 | 0.091 | 0.050 | 0.000 | 0.000 | 0.000 | 0.000 | 0.883 | 0.718 | 0.549 | 0.530 | 0.589 | 0.929 |
| C35:2_2 | 3,454 | 0.000 | 0.000 | 0.000 | 0.000 | 0.000 | 0.000 | 0.000 | 0.000 | 0.000 | 0.000 | **0.707** | **0.629** |
| 12-; 10-C35:1 | 3,470 | 0.148 | 0.099 | 0.000 | 0.000 | 0.000 | 0.000 | 1.418 | 0.945 | 0.000 | 0.000 | 0.775 | 0.545 |
| C35 | 3,500 | 0.000 | 0.000 | 0.000 | 0.000 | 0.000 | 0.000 | **0.281** | **0.340** | 0.000 | 0.000 | 0.000 | 0.000 |

Table S2: CHC profile of nurse bees from different *A. mellifera* subspecies showing mean relative abundance (%) and standard deviation (sd) of all compounds. Mean and sd values are in bold for the cases a compound is present in only one subspecies.

| Compound | RI | *A. m. carnica* | | *A. m. iberiensis* | | *A. m. ligustica* | | *A. m. macedonica* | | *A. m. mellifera* | | *A. m. ruttneri* | |
| --- | --- | --- | --- | --- | --- | --- | --- | --- | --- | --- | --- | --- | --- |
|  |  | mean | sd | mean | sd | mean | sd | mean | sd | mean | sd | mean | sd |
| C17:2 | 1,659 | 0.000 | 0.000 | **0.048** | **0.057** | 0.000 | 0.000 | 0.000 | 0.000 | 0.000 | 0.000 | 0.000 | 0.000 |
| 8-C17:1 | 1,666 | 0.000 | 0.000 | 0.178 | 0.182 | 0.000 | 0.000 | 0.000 | 0.000 | 0.020 | 0.028 | 0.000 | 0.000 |
| C17 | 1,700 | 0.000 | 0.000 | 0.277 | 0.388 | 0.000 | 0.000 | 0.000 | 0.000 | 0.028 | 0.026 | 0.000 | 0.000 |
| C19:2 | 1,863 | 0.000 | 0.000 | **0.019** | **0.023** | 0.000 | 0.000 | 0.000 | 0.000 | 0.000 | 0.000 | 0.000 | 0.000 |
| 9-C19:1 | 1,875 | 0.000 | 0.000 | 0.243 | 0.124 | 0.230 | 0.159 | 0.180 | 0.199 | 0.281 | 0.213 | 0.053 | 0.042 |
| C19 | 1,900 | 0.000 | 0.000 | 0.943 | 0.989 | 0.137 | 0.101 | 0.068 | 0.090 | 0.228 | 0.197 | 0.131 | 0.073 |
| C20 | 2,000 | 0.000 | 0.000 | 0.037 | 0.039 | 0.000 | 0.000 | 0.000 | 0.000 | 0.027 | 0.013 | 0.000 | 0.000 |
| 9-C21:1 | 2,073 | 0.000 | 0.000 | 0.115 | 0.101 | 0.000 | 0.000 | 0.000 | 0.000 | 0.046 | 0.027 | 0.000 | 0.000 |
| C21 | 2,100 | 0.000 | 0.000 | 2.137 | 1.301 | 1.426 | 1.186 | 0.626 | 0.735 | 1.118 | 0.528 | 1.206 | 0.689 |
| C22:1 | 2,173 | 0.000 | 0.000 | 0.000 | 0.000 | 0.000 | 0.000 | 0.000 | 0.000 | **0.013** | **0.016** | 0.000 | 0.000 |
| C22 | 2,200 | 0.000 | 0.000 | 0.221 | 0.098 | 0.151 | 0.052 | 0.086 | 0.079 | 0.270 | 0.086 | 0.217 | 0.114 |
| C23:2 | 2,264 | 0.000 | 0.000 | 0.123 | 0.173 | 0.000 | 0.000 | 0.000 | 0.000 | 0.129 | 0.176 | 0.000 | 0.000 |
| 9-; 7-C23:1 | 2,271 | 0.555 | 0.736 | 1.010 | 0.538 | 0.802 | 0.736 | 0.599 | 0.501 | 1.890 | 0.880 | 1.181 | 0.276 |
| C23 | 2,300 | 7.740 | 9.622 | 5.336 | 4.512 | 4.522 | 4.518 | 3.625 | 4.816 | 7.146 | 4.388 | 5.750 | 4.269 |
| 11-MeC23 | 2,334 | 0.000 | 0.000 | 0.061 | 0.078 | 0.000 | 0.000 | 0.032 | 0.057 | 0.042 | 0.044 | 0.000 | 0.000 |
| 9-C24:1 | 2,371 | 0.000 | 0.000 | 0.000 | 0.000 | 0.000 | 0.000 | 0.000 | 0.000 | **0.107** | **0.066** | 0.000 | 0.000 |
| C24 | 2,400 | 0.000 | 0.000 | 0.269 | 0.068 | 0.236 | 0.128 | 0.069 | 0.028 | 0.452 | 0.120 | 0.284 | 0.141 |
| C25:2_1 | 2,455 | 0.000 | 0.000 | 0.171 | 0.265 | 0.012 | 0.015 | 0.073 | 0.133 | 0.035 | 0.071 | 0.083 | 0.124 |
| 4-MeC24 | 2,460 | 0.000 | 0.000 | 0.000 | 0.000 | 0.000 | 0.000 | 0.000 | 0.000 | **0.018** | **0.009** | 0.000 | 0.000 |
| C25:2_2 | 2,465 | 0.000 | 0.000 | 0.043 | 0.038 | 0.078 | 0.157 | 0.000 | 0.000 | 0.105 | 0.076 | 0.000 | 0.000 |
| 9-; 7-C25:1 | 2,471 | 1.269 | 1.941 | 0.722 | 0.345 | 1.091 | 1.251 | 0.549 | 0.602 | 1.735 | 1.209 | 0.599 | 0.632 |
| C25 | 2,500 | 7.888 | 10.901 | 4.160 | 1.682 | 6.135 | 4.483 | 2.783 | 0.836 | 9.359 | 4.760 | 5.967 | 4.708 |
| 13-; 11-MeC25 | 2,533 | 0.175 | 0.315 | 0.355 | 0.415 | 0.142 | 0.057 | 0.304 | 0.529 | 0.255 | 0.327 | 0.358 | 0.359 |
| 5-MeC25 | 2,547 | 0.000 | 0.000 | 0.031 | 0.043 | 0.000 | 0.000 | 0.000 | 0.000 | 0.000 | 0.000 | 0.033 | 0.033 |
| C26:1_1 | 2,571 | 0.000 | 0.000 | 0.000 | 0.000 | 0.000 | 0.000 | 0.000 | 0.000 | **0.078** | **0.033** | 0.000 | 0.000 |
| 3-MeC25 | 2,571 | 0.000 | 0.000 | 0.028 | 0.024 | 0.010 | 0.013 | 0.027 | 0.032 | 0.000 | 0.000 | 0.042 | 0.029 |
| 9-C26:1 | 2,574 | 0.000 | 0.000 | 0.000 | 0.000 | 0.000 | 0.000 | 0.000 | 0.000 | **0.092** | **0.056** | 0.000 | 0.000 |
| 5,15-diMeC25 | 2,583 | 0.000 | 0.000 | 0.000 | 0.000 | 0.000 | 0.000 | **0.061** | **0.102** | 0.000 | 0.000 | 0.000 | 0.000 |
| C26 | 2,600 | 0.190 | 0.191 | 0.581 | 0.117 | 0.366 | 0.080 | 0.236 | 0.059 | 0.837 | 0.108 | 0.478 | 0.184 |
| 13-MeC26 | 2,630 | 0.000 | 0.000 | 0.078 | 0.080 | 0.039 | 0.011 | 0.077 | 0.094 | 0.060 | 0.063 | 0.077 | 0.086 |
| C27:2_1 | 2,643 | 0.000 | 0.000 | 0.230 | 0.378 | 0.023 | 0.024 | 0.059 | 0.105 | 0.039 | 0.050 | 0.000 | 0.000 |
| C27:2_2 | 2,649 | 0.000 | 0.000 | 0.000 | 0.000 | 0.000 | 0.000 | 0.000 | 0.000 | **0.023** | **0.027** | 0.000 | 0.000 |
| 4-MeC26 | 2,660 | 0.000 | 0.000 | **0.012** | **0.019** | 0.000 | 0.000 | 0.000 | 0.000 | 0.000 | 0.000 | 0.000 | 0.000 |
| 9-C27:1 | 2,671 | 0.201 | 0.268 | 0.343 | 0.071 | 0.350 | 0.258 | 0.238 | 0.114 | 1.519 | 0.933 | 0.351 | 0.314 |
| 7-C27:1 | 2,678 | 0.055 | 0.077 | 0.314 | 0.157 | 0.108 | 0.077 | 0.054 | 0.016 | 0.933 | 0.787 | 0.098 | 0.068 |
| C27 | 2,700 | 12.933 | 4.429 | 15.776 | 3.668 | 10.761 | 1.993 | 10.104 | 3.531 | 19.477 | 3.589 | 13.316 | 3.680 |
| 13-; 11-MeC27 | 2,727 | 1.866 | 3.246 | 2.889 | 3.679 | 1.209 | 0.910 | 2.027 | 3.295 | 1.420 | 2.345 | 3.477 | 4.438 |
| 7-MeC27 | 2,738 | 0.000 | 0.000 | 0.118 | 0.150 | 0.000 | 0.000 | 0.087 | 0.132 | 0.000 | 0.000 | 0.000 | 0.000 |
| 5-MeC27 | 2,747 | 0.000 | 0.000 | 0.047 | 0.063 | 0.000 | 0.000 | 0.045 | 0.065 | 0.000 | 0.000 | 0.062 | 0.080 |
| 11,15-diMeC27 | 2,756 | 0.000 | 0.000 | 0.121 | 0.226 | 0.000 | 0.000 | 0.112 | 0.160 | 0.000 | 0.000 | 0.000 | 0.000 |
| x,x-diMeC27 | 2,758 | 0.000 | 0.000 | 0.000 | 0.000 | 0.000 | 0.000 | **0.086** | **0.124** | 0.000 | 0.000 | 0.000 | 0.000 |
| y,y-diMeC27 | 2,762 | 0.000 | 0.000 | 0.000 | 0.000 | 0.000 | 0.000 | **0.084** | **0.122** | 0.000 | 0.000 | 0.000 | 0.000 |
| z,z-diMeC27 | 2,770 | 0.000 | 0.000 | 0.000 | 0.000 | 0.000 | 0.000 | **0.118** | **0.155** | 0.000 | 0.000 | 0.000 | 0.000 |
| C28:1_1 | 2,772 | 0.000 | 0.000 | 0.151 | 0.131 | 0.000 | 0.000 | 0.000 | 0.000 | 0.115 | 0.065 | 0.000 | 0.000 |
| 3-MeC27 | 2,772 | 0.000 | 0.000 | 0.000 | 0.000 | 0.026 | 0.034 | 0.000 | 0.000 | 0.000 | 0.000 | 0.021 | 0.026 |
| C28:1_2 | 2,781 | 0.000 | 0.000 | 0.288 | 0.324 | 0.000 | 0.000 | 0.000 | 0.000 | 0.156 | 0.065 | 0.000 | 0.000 |
| 5,17-diMeC27 | 2,780 | 0.000 | 0.000 | 0.000 | 0.000 | 0.000 | 0.000 | **0.118** | **0.115** | 0.000 | 0.000 | 0.000 | 0.000 |
| C28 | 2,800 | 0.421 | 0.207 | 1.136 | 0.236 | 0.525 | 0.123 | 0.459 | 0.216 | 0.699 | 0.130 | 0.579 | 0.353 |
| 14-; 13-MeC28 | 2,830 | 0.000 | 0.000 | 0.335 | 0.451 | 0.116 | 0.115 | 0.300 | 0.415 | 0.129 | 0.255 | 0.438 | 0.641 |
| C29:2_1 | 2,847 | 0.000 | 0.000 | 0.025 | 0.020 | 0.000 | 0.000 | 0.000 | 0.000 | 0.038 | 0.031 | 0.000 | 0.000 |
| C29:2_2 | 2,853 | 0.000 | 0.000 | 0.000 | 0.000 | 0.000 | 0.000 | 0.000 | 0.000 | **0.136** | **0.086** | 0.000 | 0.000 |
| C29:2_3 | 2,860 | 0.000 | 0.000 | 0.120 | 0.054 | 0.000 | 0.000 | 0.000 | 0.000 | 0.114 | 0.027 | 0.000 | 0.000 |
| 4-MeC28 | 2,860 | 0.000 | 0.000 | 0.000 | 0.000 | 0.000 | 0.000 | 0.045 | 0.039 | 0.000 | 0.000 | 0.087 | 0.068 |
| 10-; 9-; 8-; 7-C29:1 | 2,875 | 0.631 | 0.185 | 4.869 | 2.605 | 0.855 | 0.398 | 0.740 | 0.131 | 4.192 | 1.095 | 0.680 | 0.108 |
| C29 | 2,900 | 13.658 | 4.739 | 16.966 | 5.840 | 9.602 | 1.511 | 7.074 | 0.990 | 11.856 | 3.424 | 6.837 | 2.622 |
| 15-; 13-MeC29 | 2,928 | 2.069 | 3.428 | 1.294 | 3.195 | 1.622 | 1.500 | 3.093 | 4.182 | 0.514 | 0.311 | 4.580 | 6.964 |
| 13,17-diMeC29 | 2,951 | 0.000 | 0.000 | 0.000 | 0.000 | 0.000 | 0.000 | 0.195 | 0.252 | 0.086 | 0.102 | 0.000 | 0.000 |
| 11,17-diMeC29 | 2,958 | 0.000 | 0.000 | 0.000 | 0.000 | 0.000 | 0.000 | 0.414 | 0.516 | 0.202 | 0.276 | 0.000 | 0.000 |
| C30:2 | 2,959 | 0.000 | 0.000 | **0.626** | **0.620** | 0.000 | 0.000 | 0.000 | 0.000 | 0.000 | 0.000 | 0.000 | 0.000 |
| 10-; 9-; 8-; 7-C30:1 | 2,976 | 0.000 | 0.000 | 0.146 | 0.130 | 0.118 | 0.067 | 0.246 | 0.083 | 0.391 | 0.227 | 0.000 | 0.000 |
| 5-,17-; 5,15-diMeC29 | 2,980 | 0.000 | 0.000 | 0.000 | 0.000 | 0.000 | 0.000 | 0.000 | 0.000 | **0.058** | **0.106** | 0.000 | 0.000 |
| C30:1 | 2,983 | 0.000 | 0.000 | 0.362 | 0.212 | 0.000 | 0.000 | 0.072 | 0.040 | 0.000 | 0.000 | 0.000 | 0.000 |
| C30 | 3,000 | 0.247 | 0.140 | 0.494 | 0.150 | 0.347 | 0.050 | 0.247 | 0.032 | 0.305 | 0.091 | 0.332 | 0.063 |
| 15-; 14-MeC30 | 3,030 | 0.000 | 0.000 | 0.205 | 0.216 | 0.053 | 0.078 | 0.154 | 0.215 | 0.058 | 0.122 | 0.249 | 0.360 |
| C31:2_1 | 3,051 | 0.000 | 0.000 | 0.000 | 0.000 | 0.429 | 0.228 | 0.000 | 0.000 | 0.267 | 0.102 | 0.000 | 0.000 |
| C31:2_2 | 3,057 | 0.079 | 0.062 | 2.181 | 1.791 | 0.000 | 0.000 | 0.000 | 0.000 | 0.860 | 0.517 | 0.206 | 0.087 |
| 10-; 9-; 8-C31:1 | 3,075 | 10.199 | 4.628 | 10.752 | 5.823 | 11.378 | 3.335 | 9.924 | 3.786 | 12.345 | 4.861 | 6.134 | 3.266 |
| C31 | 3,100 | 11.501 | 5.855 | 6.727 | 3.540 | 8.826 | 1.386 | 6.147 | 2.135 | 5.313 | 2.579 | 7.316 | 4.155 |
| 15-; 13-MeC31 | 3,125 | 1.113 | 1.687 | 2.045 | 2.397 | 1.498 | 1.027 | 2.189 | 2.337 | 0.829 | 1.108 | 3.151 | 3.946 |
| 11, 17-diMeC31 | 3,149 | 0.000 | 0.000 | 0.000 | 0.000 | 0.000 | 0.000 | 0.000 | 0.000 | **0.457** | **0.303** | 0.000 | 0.000 |
| 13, 17-diMeC31 | 3,150 | 0.000 | 0.000 | 0.000 | 0.000 | 0.000 | 0.000 | **0.736** | **0.349** | 0.000 | 0.000 | 0.000 | 0.000 |
| C32:2_1 | 3,150 | 0.000 | 0.000 | **0.542** | **0.467** | 0.000 | 0.000 | 0.000 | 0.000 | 0.000 | 0.000 | 0.000 | 0.000 |
| C32:2_2 | 3,158 | 0.000 | 0.000 | 0.000 | 0.000 | 0.210 | 0.271 | 0.000 | 0.000 | 0.119 | 0.206 | 0.090 | 0.133 |
| 10-; 9-C32:1 | 3,173 | 0.517 | 0.330 | 0.562 | 0.331 | 1.005 | 0.406 | 1.172 | 0.416 | 0.343 | 0.307 | 0.749 | 0.379 |
| C32 | 3,200 | 0.000 | 0.000 | 0.226 | 0.243 | 0.270 | 0.300 | 0.232 | 0.117 | 0.176 | 0.083 | 0.285 | 0.148 |
| C33:2_1 | 3,249 | 0.872 | 0.545 | 0.000 | 0.000 | 3.329 | 1.016 | 4.565 | 2.388 | 2.314 | 1.772 | 1.850 | 2.087 |
| C33:2_2 | 3,257 | 0.000 | 0.000 | **1.852** | **1.175** | 0.000 | 0.000 | 0.000 | 0.000 | 0.000 | 0.000 | 0.000 | 0.000 |
| C33:2_3 | 3,265 | 0.000 | 0.000 | **0.606** | **0.564** | 0.000 | 0.000 | 0.000 | 0.000 | 0.000 | 0.000 | 0.000 | 0.000 |
| 10-C33:1 | 3,278 | 22.528 | 11.631 | 6.226 | 2.871 | 23.930 | 8.950 | 26.130 | 13.351 | 6.975 | 3.279 | 23.700 | 13.826 |
| C33 | 3,300 | 2.456 | 1.694 | 1.033 | 0.443 | 2.993 | 0.801 | 2.862 | 0.919 | 1.122 | 0.655 | 2.771 | 1.614 |
| 17-; 15-; 13-MeC33 | 3,327 | 0.000 | 0.000 | 1.307 | 1.777 | 1.224 | 1.185 | 2.642 | 2.111 | 0.602 | 0.489 | 2.014 | 1.630 |
| 13, 19-diMeC33 | 3,349 | 0.000 | 0.000 | 0.000 | 0.000 | 0.000 | 0.000 | 0.000 | 0.000 | **0.343** | **0.158** | 0.000 | 0.000 |
| C35:2_1 | 3,449 | 0.223 | 0.227 | 1.439 | 3.113 | 1.895 | 0.640 | 3.464 | 1.186 | 0.610 | 0.294 | 2.041 | 1.484 |
| 12-; 10-C35:1 | 3,470 | 0.614 | 0.443 | 0.449 | 0.231 | 1.921 | 0.616 | 3.770 | 1.477 | 0.495 | 0.149 | 2.129 | 1.642 |
| C35 | 3,500 | 0.000 | 0.000 | 0.000 | 0.000 | 0.000 | 0.000 | **0.406** | **0.218** | 0.000 | 0.000 | 0.000 | 0.000 |
| 19-; 17-; 15-MeC37 | 3,722 | 0.000 | 0.000 | 0.000 | 0.000 | 0.000 | 0.000 | **0.193** | **0.153** | 0.000 | 0.000 | 0.000 | 0.000 |

Table S3: One-way permutational test of multivariate homogeneity of group dispersions contrasting the variance in the CHC profiles between task performance groups. The permutations (n=1000) were restricted to the Subspecies. Df - degrees of freedom; SS - sum of squares; MS - mean of squares; F - F-value; SES - standard effect size.

|  | Df | SS | MS | F | SES | p-value |
| --- | --- | --- | --- | --- | --- | --- |
| Nurses - Foragers | 1 | 0.150 | 0.150 | 18.291 | 14.983 | 0.001 |
| Residual | 118 | 0.970 | 0.008 |  |  |  |
| Total | 119 | 1.120 | 0.159 |  |  |  |


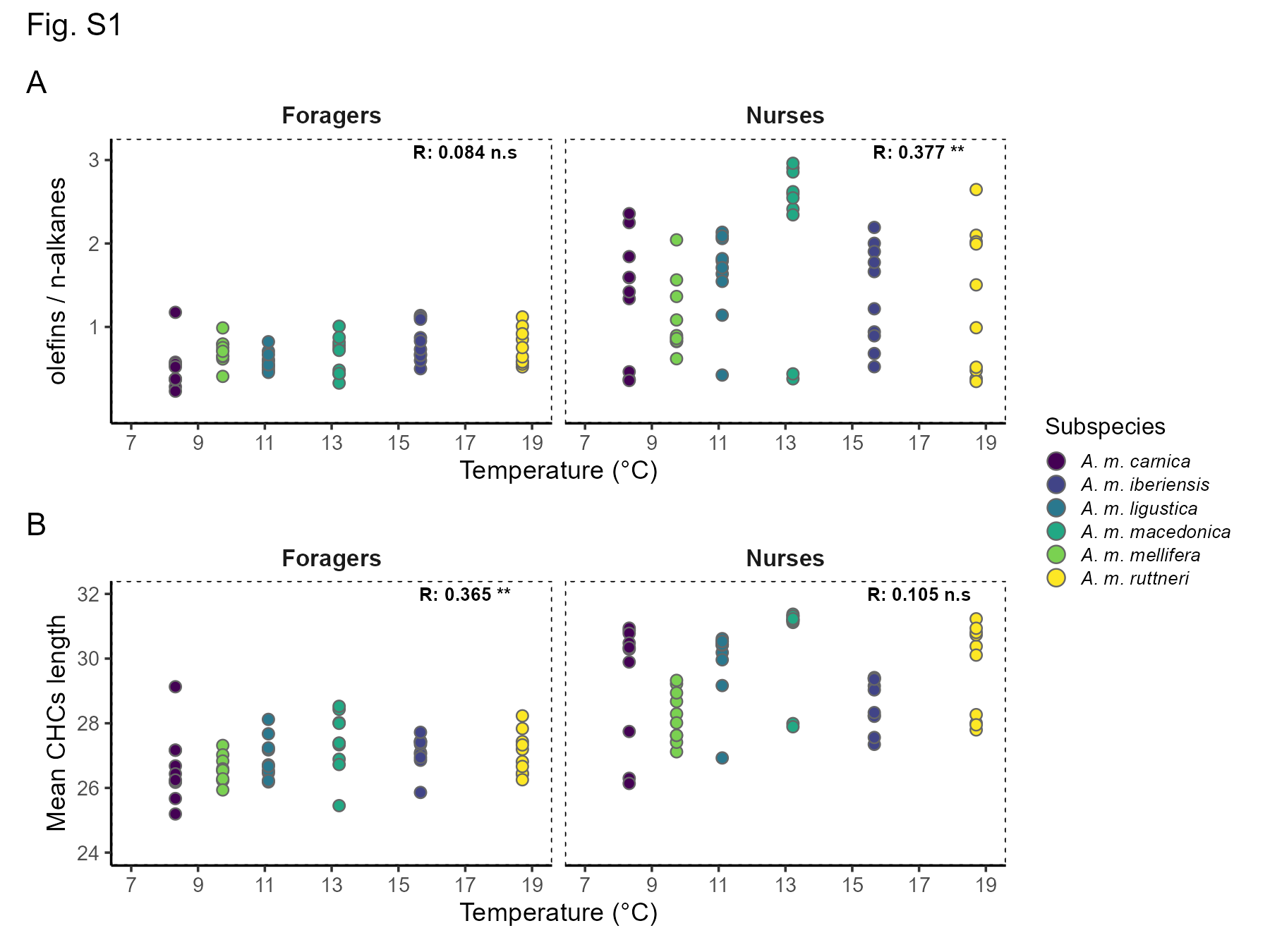


CHC composition vs temperature. A) Spearman’s correlation (R) between the olefins to n-alkanes ratio in the CHC profile of worker bees of different *A. mellifera* subspecies and the average temperature (°C) of the country of origin of the honey bee queens. B) Spearman’s correlation (R) between the weighted mean chain length of hydrocarbons in the CHC profile of worker bees of different *A. mellifera* subspecies and the average temperature (°C) of the country of origin of the honey bee queens. Significance of the correlation is indicated as n.s (p-value ≥ 0.05), * (p-value < 0.05), ** (p-value < 0.01), *** (p-value < 0.001)


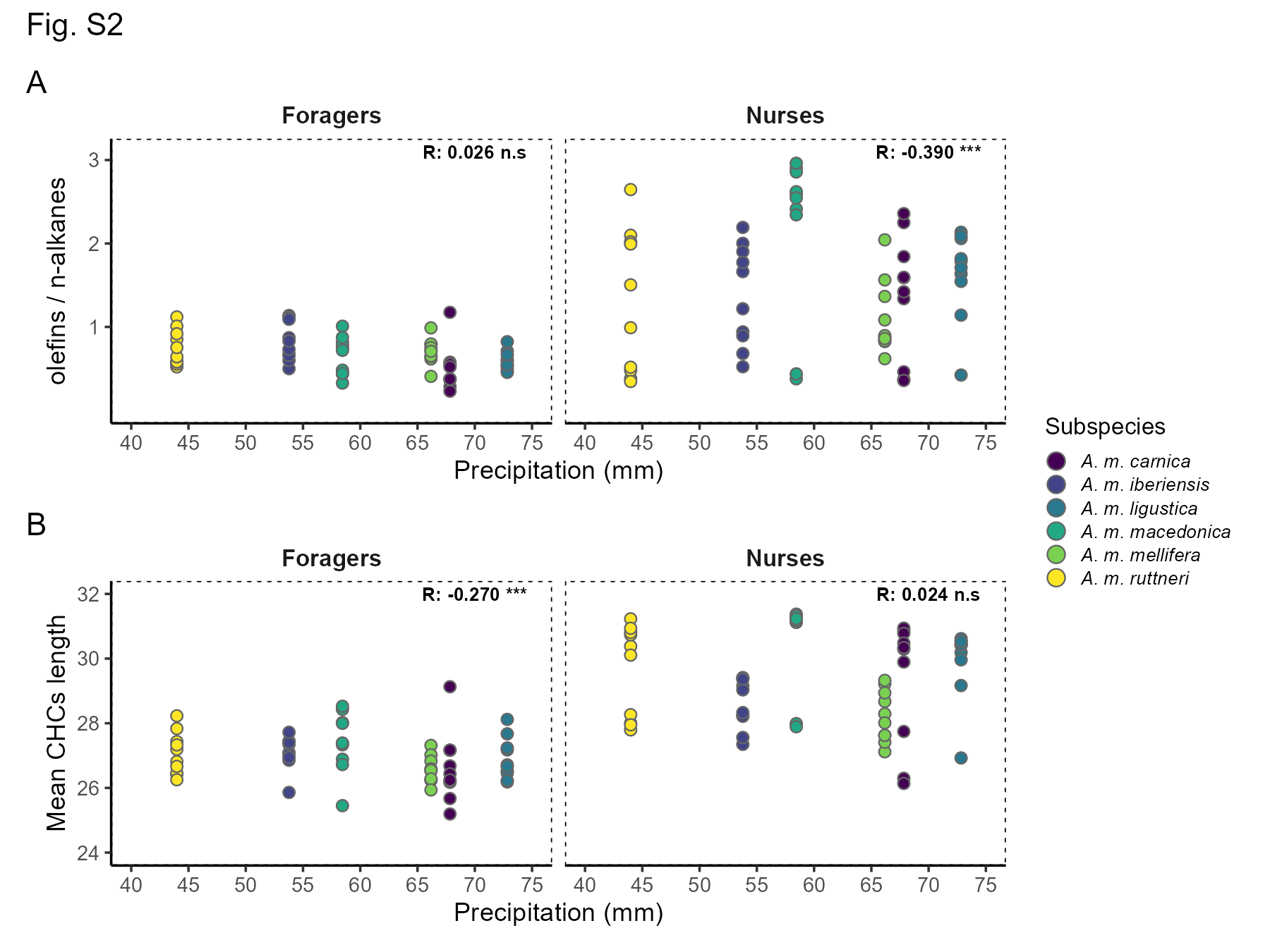


CHC composition vs precipitation A) Spearman’s correlation (R) between the olefins to n-alkanes ratio in the CHC profile of worker bees of different *A. mellifera* subspecies and the average precipitation (mm) of the country of origin of the honey bee queens. B) Spearman’s correlation (R) between the weighted mean chain length of hydrocarbons in the CHC profile of worker bees of different *A. mellifera* subspecies and the average precipitation (mm) of the country of origin of the honey bee queens. Significance of the correlation is indicated as n.s (p-value ≥ 0.05), * (p-value < 0.05), ** (p-value < 0.01), *** (p-value < 0.001)
